# Update on the Potential of *Punica granatum* L. Traditional Uses and Pharmacological Uses: A Review

**DOI:** 10.1155/adpp/6523809

**Published:** 2024-11-30

**Authors:** Abdulrahman Mahmoud Dogara, Harmand A. Hama, Dogan Ozdemir

**Affiliations:** Biology Education Department, Tishk International University, Erbil, Iraq

**Keywords:** antioxidant, ethnobotany, parasites, plant

## Abstract

Since the dawn of civilization, humans have turned to plants as a reliable source of safe and efficient treatment for a wide variety of medical conditions. The medicinal value of *Punica granatum* has been recognized for some time. Inflammation, diabetes, parasitic infections, cancer, and many other diseases have all been treated with its components. This review provides a comprehensive overview of the current biological data (those from 2018 to 2023 are included in the preclinical studies while articles of clinical studies have no limit due to their scarcity) and explores the potential applications of *P. granatum* as a novel platform for treating various disease conditions. Electronic searches for scholarly articles were performed using Elsevier, Springer, Google Scholar, Taylor & Francis, PubMed, and Scopus. Research the following terms: “*Punica granatum*,” “chemical composition,” “antioxidant,” “antibacterial,” “anti-diabetic,” “anticancer,” and other relevant terms. It has been scientifically proven that the fruit peel exhibits antioxidant, anti-inflammatory, antimicrobial, antiparasitic, antidiabetic, hepatoprotective, nerve-recovery, antihypertensive, anti-asthma, wound healing, and anticancer activities. Based on both preclinical and clinical experimentation on *P. granatum*, there is considerable evidence that supports the use of *P. granatum* extract as therapeutic agent for different ailments. The review paved the ground to precursor evidence of *P. granatum* extract benefits with its antioxidant, anti-inflammatory, antimicrobial, and antidiabetic properties. Furthermore, clinical trials stand out as a substrate supporting these effects with the enhancements of ailments including post menstrual, menstrual pain, semen quality, knee joint arthritis, and cardiovascular-related diseases. Nonetheless, more controlled large-scale clinical trials are needed for all the conditions to determine the effectiveness and risk benefit profile of *P. granatum* extract for these diseases.

## 1. Introduction

For millennia, people have used plants as medicine, first as traditional combinations and, subsequently, as pure active components, passing down their knowledge and skills from generation to generation [1]. The topic of phytochemistry is now receiving more attention and interest in manufacturing pharmaceuticals [2]. Plants are crucial to the generation of human food. Medicinal and aromatic plants (MAPs) have great commercial value due to their long history of traditional use [3].

The use of medicinal plants is the cornerstone of alternative medicine, and it has inspired the development of several cutting-edge drugs. Increasingly more than 80% of medicine in the nineteenth century was derived from plants, and because of the scientific revolution, the pharmaceutical business expanded, and products created by it became increasingly well known. Medicinal plants are utilized increasingly commonly in the treatment of ailments because they are known as safe, effective treatments with little negative effects [2].

Nowadays, people across the globe enjoy eating pomegranates, which are among the oldest fruits that can be eaten. The abundance of bioactive substances in various parts of this plant's fruits, seeds, flowers, and leaves has led to a surge of attention from scientists in recent years [4]. Realizing the potential of medicinal plants, the World Health Organization (WHO) started compiling information on their application to integrate complementary and alternative medicine into the healthcare system [5]. Pomegranate has gained global attention nowadays due to its medicinal potential. Traditionally, *Punica granatum* has been used to manage chronic diseases [6, 7]. Therefore, they could be a natural alternative to pharmacological treatment. Pomegranates have been found to have anti-inflammatory, antiviral, antibacterial, anticarcinogenic, and antidiabetic properties [8].

Previously, review papers are published on its chemical composition, biological activity, and ethnopharmacology [9–12]. The review by Valero-Mendoza et al. [13] focuses on pomegranate peel. Notwithstanding the many studies on *P. granatum* released recently, including those on its biological characteristics and therapeutic applications, there are still major gaps in the way traditional knowledge is incorporated with contemporary pharmacological discoveries. Similarly, new information is always added, so there is a need to provide up-to-date data about *P. granatum*. It is essential to analyze these species using the latest understanding of biological activity, and doing so may help to bridge the gap between accepted wisdom and research supported by evidence. Our review seeks to close these important gaps by providing a comprehensive analysis of *P. granatum* that spans modern scientific study with historical practices. This review will offer a more complete knowledge of the *P. granatum* components' full potential and efficacy by including traditional usage with recent pharmacological discoveries. Although earlier studies have made important contributions, a thorough investigation combining traditional information with biological insights is still needed to solve the theoretical and pragmatic consequences of pomegranate's health advantages. The systematic narrative review focuses on its biological activity, examines ethnopharmacology, origin and distribution, taxonomic, morphological, and biological evaluations of scientific papers published between 2018 and 2023. This review will not only compile current information but also highlight and solve discrepancies and gaps in the present literature, therefore providing fresh angles on how conventional and modern techniques might interact. This will direct next investigations and useful uses, thereby improving the usage of *P. granatum* in conventional and modern environments.

## 2. Methodology

**Figure 1:**
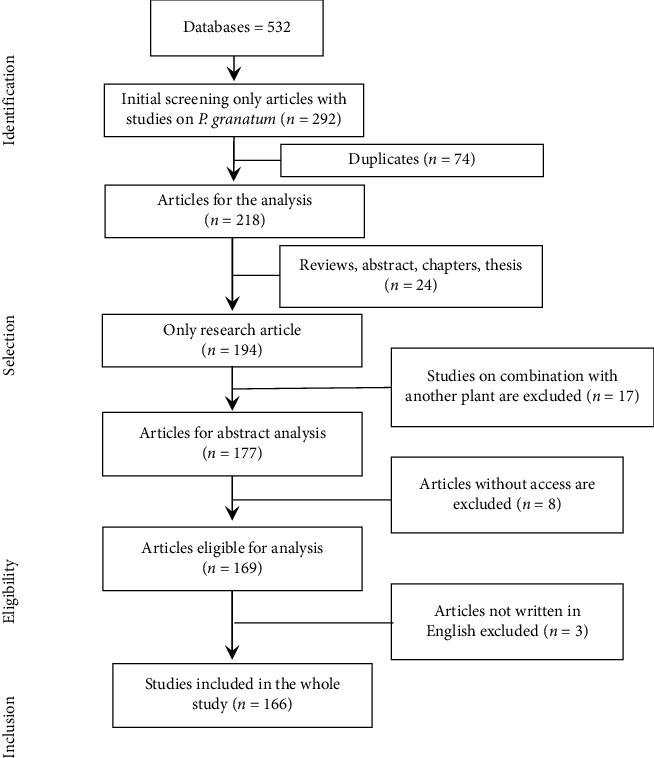
Flowchart of the methodology for article selection.

A systematic narrative literature review was carried out to identify, synthesize, and compile scientific research information on *P. granatum*. ScienceDirect, PubMed, Wiley, Google Scholar, Hindawi, and Springer retrieved useful information from original scientific research papers. Criteria for inclusion: Traditional medicine, ethnopharmacology, toxicity, cytotoxic activity, chemical composition, mineral components, gas chromatography and mass spectrometry (GCMS) analysis, pomegranate, *P. granatum*, and other related terms were used as filters to discover studies (Table 1). Only articles from 2018 to 2023 are included in the preclinical studies while articles of clinical studies have no limit due to their scarcity. Exclusion criteria: This analysis eliminated data published before year 2018, as well as thesis papers, abstract, preprint, and review publications published before 2018 (Figure 1).

### 2.1. Taxonomy and Distribution

Pomegranate is a common name for the plant *P. granatum*. The word “pomegranate” comes from the Medieval Latin words “pomum” and “granatum,” which both imply seeded [14]. *P. granatum* and *P. protopunica* Balf. make up the genus's two species. *P. granatum* is a hybrid of the Latin words punica, meaning “of or relating to Carthage,” and granatum, meaning filled with seeds [14]. *P. granatum* is an herbaceous annual, perennial, shrub, or tree of the family Lythraceae. There are 31 genera and 600-620 species in this family, and they are all found in the tropics [15]. *P. granatum* L. (Lythraceae), more commonly recognized as pomegranate, is a fruit that has been grown in the Mediterranean region since ancient times despite its native range stretching from Iran to Northern India. Fruits, whether fresh or processed into juice or jam, are a common part of many people's diets [16]. Pomegranate is often believed to be native to the area spanning from Iran to Northern India, where it grows wild in many woodlands, but is a naturalized species in the Mediterranean and North African regions [15]. The pomegranate, or *P. granatum*, is a tiny tree of the family Lythraceae [17]. The Mediterranean countries of Persia, Turkey, Egypt, and Spain, as well as California, China, Japan, and Russia, have all benefited from its cultivation [17].

### 2.2. Morphology and Anatomy

**Figure 2:**
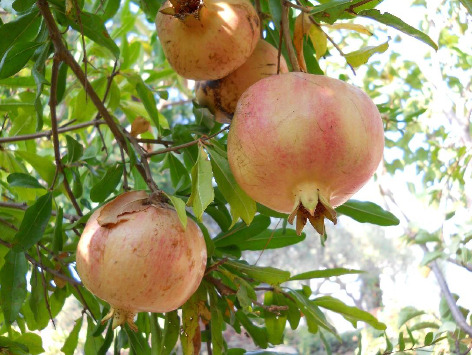
*Punica granatum* (https://www.vdberk.com/trees/punica-granatum/).

A pomegranate is a prickly shrub or small tree (height 2–7 m) with scarlet red (occasionally white) blooms (Figure 2) [15]. The plant is a tiny, deciduous tree with occasionally prickly branches, simple oblong, or obovate leaves (Figure 1), an actinomorphic inflorescence, bisexual red flowers with many stamens and a gamosepalous and polypetalous condition, and an inferior ovary with axile and parietal placentation [14]. The outermost layer of a leaf's epidermis has thick walls and is practically square in shape, as seen under a microscope. There are a few cells in the top epidermis whose lateral outlines are slightly undulating [18]. The cells in the lower epidermis are longer and more polygonal than those in the upper layers, and their membranes are thicker and more curved [18]. The stomata were only found in the basal layer of the skin [18]. There were a lot of them, and their shape was elliptical. Up to five amniocytic respiratory complex cells can be seen in the peritoneal cavity [18]. The leaf blade (Figure 2) is organized dorsally in a cross section. While both the upper and lower epidermis consist of a single layer, the cuticle that covers the upper epidermis cells is significantly thicker and smoother than that which covers the lower epidermis cells [18]. Epidermis and cuticles were easily discerned in a transverse piece of a fruit rind [19]. Vascular bundles, crystal-like ergastic inclusions, and small needle-like structures make up the ground tissue [19]. The area was found to be riddled with fiber aggregates. The abundance of stone cells is a defining microscopic feature of this region. The transverse section of *P*. *granatum* immature seeds revealed sclerenchyma cells grouped peripherally around the embryo. The clear, well-defined endosperm was found only in the core part of the seed, where it appeared to be restricted like a nucleolus [19].

### 2.3. Traditional Uses

Pomegranate (*P. granatum*) has been studied extensively for its purported health benefits and has a storied medical past. *P. granatum* has been employed in alternative medicine for the cure of a wide range of conditions, including the removal of tapeworms [20]. Pomegranate fruit, peel, and root are commonly used in herbal medicine in many countries. To boost your immunity against any form of viral infection, consume the extract of pomegranate daily [6, 7, 21]. It is recommended to gargle or drink extract made from the pomegranate peel [21]. Studies on ethnobotany indicate that fruits are mostly used to treat digestive problems, dry coughs, and urinary problems [22]. Traditionally, *P. granatum* has been reported to be effective in treating digestive problems [23]. *P. granatum* was identified by the interviewees as a plant used to treat diarrhea and cough [24]. The study supported the widespread usage of *P. granatum* fruit and bark as food and medicine [25]. We give a summary of how *P. granatum* species have traditionally been used to create evidence-based medications.

### 2.4. Biological Activity

**Figure 3:**
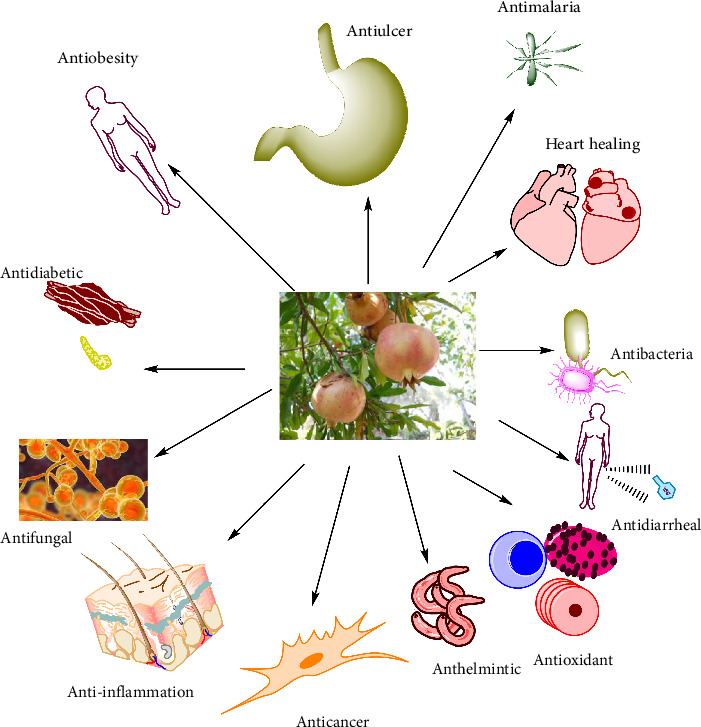
Some diseases are treated with different parts of *Punica granatum* (source: authors).

The investigation discovered that each biological evaluation of the plant's parts employed a separate assay to confirm the plant's efficacy (Figure 3 and Table 2).

#### 2.4.1. Antioxidant Activity

Finding reliable sources of antioxidants is crucial because free radicals are thought to contribute to the development of cancer, diabetes, and cardiovascular disease in humans [29]. The 2,2-diphenyl-1-picrylhydrazyl (DPPH)⁣^∗^ and 2,2′-azino-bis(3-ethylbenzothiazoline-6-sulfonic acid) (ABTS)⁣^∗^ + assays are the most used because of their sensitivity, speed, and utilization of stable radicals to measure antioxidant activity [28]. Antioxidant activity was measured using the technique, and it was shown to vary among extracts and fractions; this could be due to changes in their profiles [28]. *P. granatum* antioxidant capacity was evaluated using animal models, DPPH, ABTS, and numerous other methods (Table 2).

When compared to the standard ascorbic acid (100 μg/mL), the radical scavenging effectiveness of the aqueous extract (87.55% 4.96) was found to be significantly lower [19]. The acetonic and methanolic fractions of pomegranate also showed remarkable antiradical efficacy against the radical, with 86.9% and 79.4% radical scavenging activity, respectively [29]. The maximum DPPH⁣^∗^ radical scavenging activity (1.64 μg/mL, *R*^2^ = 0.9999) was found in the ethyl acetate fraction, which is consistent with its greater phenolic/flavonoid concentration [28]. With EC_50_ values of 4.64 and 3.63 μg/mL for DPPH and ABTS free radicals, respectively, the extract demonstrated a good overall antioxidant potential of 23.53 mg AAE/g DW [41]. A 50% scavenging dose of extract (IC_50_) of 1.715 μg/mL indicated significant antioxidant activity [26]. In the DPPH and ABTS radical scavenging assays, the extract showed an IC_50_ value of 15.602 and 724.323 μg/mL, respectively [27]. Scavenging activity against 1,1, diphenyl 2,2, picrylhydrazyl was measured at IC_50_ values of 471.7 and 509.16 μg/mL for the aqueous and ethanolic extracts, respectively. Similarly, the IC_50_ values for the destruction of hydrogen peroxide in aqueous and ethanol extract were found to be 488.76 and 478.47 μg/mL, respectively [30].

Of all the samples, peel extracts had the highest level of antioxidant activity, with three out of four extracts displaying almost 90% activity [31]. Saturation of free radical scavenging activity was measured by DPPH and ABTS at increasing doses. The highest levels of DPPH and ABTS free radical scavenging activity were found in the peel, followed by the pulp and then the seeds. If we set the threshold at *p* ≤ 0.05, then the findings are significant [32]. It suppressed the numerous signaling pathways triggered by OV more effectively than DEX. These included ROS, WNT/-catenin, and AKT. Subsequently, oxidative stress and mucin secretion were lowered, as were levels of proinflammatory (COX-2, NO, and IL-13) and profibrotic (COL1A1) mediators [33]. The pomegranate rind extract with the highest radical scavenging power was the methanol extract (88.57), followed by the water extract (87.19) [34]. The hydroalcoholic and aqueous extracts' respective IC_50_ values for their antioxidant capacity were 32.4 and 35.12 mg/mL. The aqueous extract had the lowest activity, as shown by the DPPH test (IC_50_ = 14.15 mg/mL).

The aqueous extract has the highest hydrogen peroxide trapping percentage (45.97%) [35]. Compared to ascorbic acid, which has 22.07% antioxidant activity, greater concentrations of AgNP, such as 500 μg/mL, have 25.78% [36]. All the extracts tested here showed negligible radical scavenging efficacy against the DPPH radical. The IC_50_ ranges from 0.01303 to 1.729 μg/mL, indicating that the peel extracts do have some level of radical scavenging action [116]. The membrane (87.03 percent for Valenciana and 81.95% for Wonderful) and the peel (87.55 percent for Valenciana and 89.97% for Wonderful) had the highest values [37]. An extract concentration of 2000 ppm was shown to be optimal for retarding lipid oxidation in sardine fish oil. Extract at 2000 ppm was as likely to have antioxidant potential as BHA at 200 ppm [38]. The maximum concentration of total phenolic components (230.8 mg GAE/g extract) was found in the aqueous extract. The antioxidant activity of pomegranate water extracts was very noticeable because of the fruit's high total phenolic content [39]. Ethanol extract at 250 μg/mL had the highest DPPH radical scavenging activity with an inhibition of 90.08 μg/mL [40]. These variations in the data could be attributed to the growth conditions, the analytical techniques employed, the solvents, and the plant component used. The high antiradical action of pomegranate extract may be related to its polyphenolic chemical constituents, such as gallic and ellagic acids [29].

There are phenolic bioactive components in pomegranate extract, and these chemicals likely work synergistically against free radicals, explaining the extract's demonstrated antioxidant capacity [41]. These results suggest that pomegranate's total phenolic compounds play a significant part in the fruit's antioxidant action. The pomegranate fruit's overall phenolic, anthocyanin, and flavonoid content is linked to its scavenging ability [117]. One of the possible methods of action is due to the hydroxyl groups present in the phenolic aromatic rings, and the natural polyphenolic compounds found in pomegranate extracts demonstrate their antioxidant properties [29]. These polyphenols demonstrate their antioxidant action by neutralizing free radicals, dissolving peroxides, and serving as quenching agents against singlet and triplet O_2_ because of their redox characteristics [118]. The analyzed study findings revealed the fruit peel of pomegranate extracts as a strong antioxidant, with certain extracts showing 90% efficiency of scavenging free radicals. Despite aqueous extract ability to trap hydrogen peroxide, the acetone and methanol extracts were more active at scavenging radicals. Upcoming study ought to focus on determining the precise compounds responsible for this antioxidant activity in the fruit peel of pomegranate. More research on the molecular mechanism of the how the compounds combine and interact may help us better understand their impact on oxidative stress.

#### 2.4.2. Anti-Inflammatory Activity

Inflammation is the immune system's reaction to hostile microorganisms that injure an organism's cells and vascularized tissues, such as viruses, bacteria, poisonous substances, or physical trauma. Given the significant increase in the number of inflammatory pathologies and the adverse effects of synthetic anti-inflammatory medications, many researchers from across the world are collaborating to find molecules of vegetable origin that can alleviate these bad characteristics. There has been a long-held belief that medicinal plants can aid in the drug discovery process, and this belief persists today. These plants are easily accessible and have low costs, and their utility is based on tradition. Methanolic extract at 500 μg showed the highest percentage of inhibition (71.24%), followed by aqueous extract at the same dose (68.49%) [42]. The H-scores for each treatment group were 213.23 (DSS group), 243.81 (normal group), 226.10 (240 mg/kg/d pomegranate peel extract), 238.84 15.81 (480 mg/kg/d pomegranate peel extract), 227.47 (aspirin), and 224.01 (ellagic acid) [43]. After 6 h of carrageenan injection, the effects of the methanolic and aqueous extracts displayed a significant suppression (*P<* 0.001) of the mouse paw edema in a dose-dependent approach as compared to the control group. After 6 hours, the percentages of methanolic and aqueous extracts that inhibited edema were 80.72% and 51.94%, respectively [46].

The chemical has an enzyme activity of 78.48% compared to the typical inhibitor's 89.87%. This finding demonstrates that the isolated chemical effectively inhibits LOX enzyme activity [44]. Ethanol extract of pomegranate rind demonstrated anti-inflammatory and prostaglandin-synthesis-inhibiting effects at doses of 20, 40, and 80 mg per kilogram of body weight (BW) [45]. The presence of polyphenolic components including hydrolyzable tannins and flavonoids in the fruit can partially account for the extract's anti-inflammatory action [46]. The extracts' anti-edematous efficacy in the latter stages would suggest that, like NSAIDs, they would suppress prostaglandin synthesis, including COX, in the same way. This may shed light on the route of action of the active compounds present in *P. granatum* fruit peel extracts [46].

*P. granatum* extracts, both methanolic and aqueous, have demonstrated strong anti-inflammatory properties. The methanolic extract shows a 71.24% inhibition at 500 μg, while the aqueous extract shows a 68.49% inhibition. After 6 hours of dosing, the methanolic extract achieved an 80.72% reduction in edema and the aqueous extract a 51.94% reduction. These extracts also considerably reduced edema. The anti-inflammatory benefits that have been found are ascribed to the presence of polyphenolic substances, such as hydrolyzable tannins and flavonoids, which inhibit the formation of prostaglandins. More research is needed to determine the particular molecular pathways and processes that pomegranate peel extracts use to reduce inflammation, which will help us better comprehend this mechanism. Clinical trials must also be conducted to determine the correct dosage and long-term safety of methanolic and aqueous extracts in order to ensure their efficacy and safety over time. Establishing standardized preparation methods and quality control processes for these extracts is also necessary to ensure consistent and effective treatment outcomes.

#### 2.4.3. Antibacteria Activity

Various biological mechanisms, such as changes in membrane permeability, genetic mutations, alterations in physiochemical properties, and efflux dynamics within the targeted microorganisms, have been associated with the emergence of antibiotic resistance in bacteria following extended use of synthetic antibiotics [119]. As resistance to antibacterial drugs becomes an increasingly pressing issue worldwide, scientists are on the lookout for new chemicals that could serve as therapeutic starting materials. Alternative synthesis techniques that are eco-friendly and do not employ toxic materials are required because of these emerging problems.

When tested against CNS-37 and *Staphylococcus aureus* Strain 18, the methanol extract demonstrated the greatest inhibitory zones. The minimum inhibitory concentration (MIC) ranged from 5000 to 6500 μg/mL. Methanol extracts showed the highest levels of antioxidant activity. Therefore, the pomegranate flower extracts have shown strong antioxidant and antibacterial activity against the tested mastitis pathogens [15]. Methanolic fraction of the extract showed significant activity against *S. aureus*, methicillin-resistant *S. aureus* (MRSA), *Escherichia coli*, and *Salmonella typhimurium*, with respective inhibition zones of 23.7, 21.8, 15.6, and 14.7 mm. The methanolic fraction of the extract showed a MIC of 0.125 mg/mL and a minimum bactericidal concentration (MBC) of 0.250 mg/mL against *S. aureus* [29].

The extract and fractions were more effective against Gram-positive bacteria. For Gram-negative bacteria, ethyl acetate fractions demonstrated the highest activity against *Serratia marcescens* (MIC = 7.81 μg/mL), but for *E. coli, Klebsiella pneumoniae*, and *Pseudomonas aeruginosa*, the MIC was over a thousand times higher [28]. The growth of all bacteria in the test was slowed or inhibited by all the samples. Eighteen out of twenty-four studies (four samples, and six microorganisms investigated) yielded MIC values (2.7 or 0.3 mg/mL) [31]. The most effective method of preventing *E. coli* growth was a methanolic extract of pomegranate rind, which reduced the bacteria's population by 24.00 μm. Its growth was dramatically slowed by 19.67 mm when exposed to an aqueous extract of pomegranate rind [34]. AgNP was shown to have no discernible impact on the viability of two test organisms at concentrations of 10 and 25 μg/mL. The percentage of bacterial cells that were viable in both bacteria significantly decreased over 50 μg/mL of AgNP [36]. The zone of inhibition (ZOI) was 20 and 35 mm against *Salmonella typhi* and *E. coli*, respectively. MICs against *S. typhi* and *E. coli* were 6.25 and 12.5 μg/mL, respectively [47].

There was a wide range of antibacterial activity shown in bacterial assays using crude extracts of peel, with the ethanolic extract producing the largest mean inhibition zone, at 36 ± 1 mm in diameter against MRSA. Clearance zones were found to be greatest in ethanolic extract, then aqueous, and finally chloroform [48]. ZOIs measured in millimeters were 26, 10, and 9 for *S. aureus, S. Typhimurium*, and *E. coli*, respectively, when exposed to a methanolic peel extract (50 μL) of yellow *P. granatum* extract. Against *S*. *aureus*, *S*. *Typhimurium*, *Salmonella dysenteriae*, and *E. coli*, the methanolic extract of red *P. granatum* (100 μL) showed 27, 8, 12, and 15 mm ZOI, respectively [120]. Both *Candida albicans* (MIC = 25 μg/mL; minimum fungicidal concentration (MFC) = 50 μg/mL) and *Candida krusei* (MIC = 12.5 μg/mL; MFC = 50 μg/mL) were shown to be susceptible [50]. Clinical isolates of *P. aeruginosa* and *S. aureus* were inhibited by ethanolic extracts of pomegranate seed and peel. Pomegranate seed and peel extracts both had MICs of 25 mg/mL and 12.5 mg/mL, respectively. Pomegranate peel and seed extracts were also shown to have MBCs of 25 and 50 mg/mL, respectively, making them effective against bacteria [51]. With increasing content from 10 to 30 wt.%, films displayed clear antibacterial activity against *S. aureus*, as evidenced by a significant (*P* < 0.05) expansion of the observed inhibitory zone [56]. *Bacillus subtilis* ATCC6633, a Gram-positive bacterium, was inhibited by PAgNPs with a 40 mm zone, while *E. coli* ATCC 25922, a Gram-negative bacterium, was inhibited by PAgNPs with a 30 mm zone [72]. All extended-spectrum beta-lactamase (ESBL)–producing Enterobacteriaceae were inhibited by extracts, and the strongest inhibitor had a MIC of 512 μg/mL. Particularly effective against *K. pneumoniae*, *Citrobacter freundii*, and *E. coli* are pericarp extracts. However, against MRSA species, the *P. granatum* extract (pericarp and juice) demonstrated broad-spectrum antibacterial activity with an inhibitory diameter zone size of 11 0.9 mm to 29 1.12 mm [62].

After 48 h of incubation, the data showed that the inhibitory zone around the nano-encapsulated was greater (*P*<0.05) against the tested bacteria [54]. The extract was effective against all species tested at an inhibitory concentration of 25 mg/mL to 100 mg/mL, except for *Lactobacillus casei* var Shirota [55]. When mice were given charcoal meals, ethyl acetate fractions at 100, 200, and 400 mg/kg dramatically slowed down digestion [57]. *P. granatum* combined with sodium hypochlorite and CHX produced the best effects of any group, whether the irrigants were administered singly or in combination, with maximum mean ZOIs of 23.9 and 25.7 mm, respectively [59].

The MIC for *Enterococcus faecalis* is 15.62 μg/mL. Both bacteria had MICs of 125 μg/mL [60]. The MICs and MBCs for *P. granatum* ranged from 125 to 250 and 500 to 1000 μg/mL, respectively, indicating moderate activity [61]. The MIC study showed that the prepared mouth rinse was effective against all the selected species at a concentration of 0.2% [63]. Extraction efficacies were comparable to nystatin (nearly 100% inhibition) after 14 days [121].

The inhibition zone for *Salmonella epidermidis* and *S. aureus* ranged from 13 to 19 mm, indicating that the extract showed moderate bacterial activity. To a lesser extent, the extract inhibited the growth of *E. coli*, *K. pneumoniae*, and *E. faecalis*, but it did not affect *P. aeruginosa* [64]. All the oral pathogens tested at high MIC levels showed strong antibacterial action. The effectiveness of AgNPs in decreasing bacterial numbers increases with the concentration of the particles. Green production of silver nanoparticles (AgNPs) using *P. granatum* peel extracts may provide a novel element for medications to combat oral microorganisms [65]. The reduction in oral bacteria colony count was statistically significant at *P*≤0.001 across all groups [66]. After 1 and 3 h of treatment with doses of 75 mg/mL of the extract, bacterial growth of all three tested strains was completely inhibited [67]. The scores for fecal consistency, dehydration, and depression all decreased significantly by day 6 (posttreatment) across all groups, with the highest recovery happening in group IV (T3). Treatment group IV (T3) showed the greatest improvement after 6 days (posttreatment) as measured by an increase in serum IL10 concentration, a decrease in serum TNF concentration, and a rise in serum INF concentration [68].

With a MIC of 90, 80, 80, and 60 g mL^1^ for *P. aeruginosa*, *E. coli*, *Bacillus subtilis*, and *S. aureus*, respectively, the SNP-B demonstrated improved antibacterial activity [122]. Results from the solid-medium assays show that all the extracts examined can suppress Fusarium oxysporum growth, with MIC values ranging from 1.2 mg/mL to 4.2 mg/mL [70]. At doses of 5 and 10 mg/mL, respectively, the extract's inhibition of poisonous acne-causing bacteria was 4.07 and 9.95 mmol/L [73]. *S. aureus* methanolic seed extract and aqueous seed extract were detected at a maximum inhibitory concentration of 12 mg/mL. Crude pomegranate extracts at a dosage of 12 mg/mL showed the greatest MIC against *Salmonella epidermis*. The 12 mg/mL concentration of methanolic rind extract and aqueous rind extract showed the highest MIC against *P. aeruginosa* and *Candida albicans* [123].

Previous studies have shown a correlation between the antibacterial activity of pomegranate fruit and peel extracts and their total polyphenol concentration [56]. Polyphenols found in pomegranate peel have antibacterial effects. One of three possible methods by which these polyphenols function includes (a) precipitating proteins in the cell membrane, which results in cell lysis; (b) blocking microbial enzymes by reacting with their sulfhydryl groups; or (c) interfacing with proteins in an unspecific manner. Similar to how phenolic substances can prevent microbial development by interacting with protein sulfhydryl groups to produce phenolic toxicity [56], it was also hypothesized that their effectiveness is probably due to their capacity to precipitate proteins, which causes membrane permeability in bacteria, cell lysis, and ultimately cell death. Phenolic chemicals change the permeability of microbial cell membranes, causing more molecules like ribose and Na glutamate to flow out [62].

As a result of their impacts on electron transmission, nutrition, enzymatic activity, protein, and nucleic acid synthesis, and contact with membrane proteins, the morphological and chemical integrity of the membrane may also be jeopardized [124]. Extracts of *P. granatum* have been shown to have antibacterial properties, and this may be due to the presence of phenolic chemicals, the effects of which may operate via several different pathways [15]. The cell wall could be weakened, its composition could be altered, the cytoplasmic membrane could be disturbed, membrane protein could be degraded, and membrane-integrated enzymes may be inhibited [15]. Impairs protein transport and mitochondrial function in eukaryotes, affects DNA and RNA synthesis, alters fatty acids and phospholipids, and disrupts energy production and metabolic pathways [15]. Silver nanoparticles made through green synthesis may have entered the cells and caused intracellular loss, which results in cell death, which could explain the activity. As a result of the high amounts of lipopolysaccharide (LPS) and thick peptidoglycan layer, bacteria lyses became extremely susceptible to PAgNPs [125].

The primary active chemicals responsible for this effect include polyphenols, tannins, and flavonoids. However, when there is a complex mixture of bioactive chemicals present, isolating a single ingredient responsible for the antibacterial action is challenging. That is why antibacterial actions may result from a synergistic effect of different bioactive substances that kill bacteria in different ways. In conclusion, the mechanism of action may be chemically specific to the active chemicals, and their antimicrobial activity may not be due to a single mechanism but rather to a series of events affecting the entire bacterial cell. Significant variations in the antibacterial efficacy of the different extracts may be attributed to the distinct phytochemical composition of the different parts of the plant. The study's main findings showed that pomegranate methanol extracts displayed significant antibacterial activity, particularly against Gram-positive bacteria like *S. aureus* and MRSA. The peel and rind extracts demonstrated significant inhibition zones and effective minimum MICs against multiple bacterial strains, with the methanol fraction exhibiting the highest degrees of activity. To overcome antimicrobial resistance, more research is needed to determine the phytocompounds that underlie *P. granatum* antibacterial and antifungal properties, as well as to understand how they work and how they combine with other natural and synthetic antimicrobials in a synergistic manner. *P. granatum* extract may be a promising source for the identification and development of novel antimicrobial active compounds because of the current exploratory findings. The plant could be utilized to create antibiotics that are effective against microbial illnesses.

#### 2.4.4. Antifungal Activity

Both inflammation and *Candida* colony counts were reduced with *P. granatum* gel [53]. After 24 h of exposure, the growth inhibition zone was 2.11 mm smaller than the control, and after 48 h, it was 0.83 mm larger [52]. After 24 h of exposure, the experimental group's zone of suppression of growth of *P. vulgaris* was greater than the control group by 17.22 mm, and after 48 h, by 18.44 mm [52]. The application of the oil at different concentrations on the first and tenth days fully prevented the growth of *Aspergillus niger* [58]. The MIC for *Candida albicans* is 62.50 μg/mL [60]. The mycelial growth of plant pathogens was inhibited by oils extracted from pomegranate seeds, compared to the control. The mycelial growth of *F. oxysporum* f.sp. *lycopersici* was decreased by 21.38 percent at the highest concentration tested (1000 ppm) [69]. At 50 mg/mL, MAEG inhibited *Rhodotorula* sp., with inhibition zones measuring 29.50.5 mm in diameter. In addition, the MIC range for this extract against *Rhodotorula* sp. was 0.39–0.195 mg/mL, with the MFC being more than 0.39 mg/mL [71]. The number of *Candida* colony-forming units decreased from pre- to posttreatment in both groups. Punicalagin was palatable and well-tolerated [74].

#### 2.4.5. Anthelmintic Activity

Helminthiasis is one of the most significant animal diseases in the world because it causes large output losses in grazing animals. The disease is more prevalent in developing nations because of inadequate management and control efforts. For successful helminthic care, a multimodal approach that includes both long- and short-term anthelmintic treatment is required. An increase in anthelmintic-resistant parasites has prompted research on natural product controls (Table 2). At all dose levels, the crude extracts demonstrated potential anthelmintic activity in a concentration- and time-dependent manner. The strongest nematocidal activity was produced by the extract with the highest concentration (10 mg/mL) when compared to the negative control [20]. Mortality of worms occurred at 15, 14, and 13 h of exposure to doses of 25, 50, and 75 mg/mL of the ethanolic extract causing total stoppage of movement after 9.5, 8.5, and 7.5 h of exposure [75]. *P. granatum* macerate was found to have a 50% anthelmintic impact [76]. Both the alcoholic and water-based extracts showed significant efficacy against the lice. About 46%, 27%, and 51% of rats treated with alcoholic and aqueous extracts of *P. granatum* and minoxidil, respectively, experienced the transition from the telogen to the anagen phase of the number of anagen hair follicles [77]. The extract at 100 mg/mL showed 43.01% activity, that at 150 mg/mL showed 100% activity, and that at 200 mg/mL showed 50.9% activity [78]. Extracts at 150,200 mg/mL showed considerable anthelmintic action. Hydroalcoholic extract showed maximum activity (paralysis and death of earthworms) at a concentration of 200 mg/mL, with a dose-dependent effect and suppression of spontaneous movement. The results of the current studies demonstrate the veracity of the ethnomedical claim that this plant has anthelmintic effect [79]. At a maximum dosage of 150 mg/mL, both extracts show notable anthelmintic action. Wagh, Devdhe, and Chandak showed that at 50, 100, and 150 mg/mL, respectively, paralysis occurs in 34 to 36, 30 to 32, and 28 to 30 min, and death occurs in 80, 75, and 66 min [80]. Research on extracts from *P. granatum* has shown that it has strong anthelmintic qualities. At concentrations of 150 mg/mL and 200 mg/mL, the hydroalcoholic extract showed notable effectiveness, achieving 100% activity. Furthermore, depending on the dosage, worm death occurred at varied exposure times for the ethanolic extract, which showed significant nematocidal action. At different phases of the hair follicle transition, the efficacy of the alcoholic and aqueous extracts against lice varied. To confirm the anthelmintic potential of *P. granatum* extracts, controlled research and clinical trials should be conducted in the future. Additionally, examining the mechanisms of action and refining extraction techniques may contribute to the creation of new, efficient helminthiasis treatments.

#### 2.4.6. Antiviral Activity

Since the beginning of human civilization, plants have been employed for a variety of therapeutic purposes and are a rich source of secondary metabolites. Natural substances derived from plants have made significant contributions to the area of medicine, from ancient times to the present period of research. Antiviral medications are used to stop the operation of viral functional proteins such as major protease (Mpro) [126]. This protein contributes to the cleavage of polypeptides during viral assembly in host cells. Lopinavir and ritonavir are two antiviral medications that have been tested on SARS-CoV-2 infection for Mpro inhibitors; however, neither one is yet performing up to expectations in preventing infection and viral pathogenesis [126]. As a result, there is a need for a prospective antiviral medication with comparable effectiveness or enhanced potential to the current antiviral. The primary source of promise for the development of antiviral medicines continues to be phytochemistry. Globally intensive research is currently being conducted on the creation of medicines using natural ingredients. The human herpesvirus 3 (HHV-3) may be susceptible to inhibition by an aqueous extract of *P. granatum* leaf. The phytochemicals of *P. granatum* interacted with the active site of HHV-3 protease, as determined by in silico docking [81]. Quantitative polymerase chain reaction (qPCR) and HCV NS3 protein expression level in the transient transfection model showed that P4 (IC_50_ = 28.50.02 g/mL; IC_25_ = 16 μg/mL) and P11 (IC_50_ = 72 μg/mL; IC_25_ = 41 μg/mL) significantly inhibited HCV replication [127]. The MIC for the HHV-3 isolated from chickenpox was 15.625 μg/mL, while the MIC for the HHV-3 isolated from zoster was 31.25 μg/mL. The aqueous extract was more effective than the ethanolic extract in preventing the HHV-3-caused CPE [128]. Significant antiviral activity of *P. granatum* extract has been shown, especially against HHV-3. When it comes to reducing cytopathic effects, the plant's aqueous extract works better than its ethanolic extract. Based on in silico docking experiments, these compounds bind to the HHV-3 protease, suggesting the possibility of an inhibitory action. The aqueous extract significantly inhibited HCV replication in quantitative studies, exhibiting lower half maximal inhibitory concentration (IC_50_) values than other extracts. Future research ought to concentrate on enhancing the extraction methods and extract concentrations of *P. granatum* extracts to bolster their antiviral properties. Moreover, further investigation is required to assess their effectiveness in clinical contexts and understand the mechanisms behind their antiviral activity. Research into *P. granatum* is necessary to identify and isolate potent antiviral compounds.

#### 2.4.7. Anti-Periodontitis Activity

Periodontitis is an inflammation of the tissues that support the teeth [129]. The principal etiological agent for the disease's beginning and progression is dental plaque dysbiosis, which contains bacterial species whose virulence components induce reversible or irreversible damage to the teeth's supporting connective tissue fibers and alveolar bone [129]. After the irritants are removed, gingivitis can be treated, but if the infection spreads to the periodontal tissues nearby, it can lead to periodontitis, an irreversible damaging disorder. Even though systemic antibiotics may manage and eradicate a wide variety of diseases, they have several negative side effects on patients, such as the emergence of bacterial resistance. At the 21-day and 45-day follow-ups, Group I demonstrated a greater reduction in plaque index and gingival index compared to Groups II and III. Comparing groups on pocket probing depth showed similar outcomes, with the greatest decrease occurring in Group I between Day 21 and Day 45 (*P*>0.001). Analysis of relative attachment loss showed improvements in all three groups from baseline to 45 days, with the greatest improvement seen in Group I. Improvements in Group III were not statistically significant (*P*<0.001) [130]. By the end of the research, the extract had reduced CAL, PPD, and BOP compared to the baseline, showing improvement [129].

#### 2.4.8. Wound Healing Activity

Due to inadequate sanitation, wound infection is a widespread health problem in third-world countries [131]. Injuries that cause a breach in the skin are called wounds, and the correct approach to healing wounds is necessary for restoring the skin's anatomical continuity and its damaged functional condition [132]. The stages of wound healing include coagulation, inflammation, granulation tissue creation, matrix production, connective tissue remodeling, collagenization, and acquisition of wound strength [131]. Maximum tension, swelling index, and water vapor permeability (*P*≤0.001) were all improved in membranes treated with *P. granatum* extracts [26]. In comparison to the control group, the 2% (w/w) SNP-B treated group showed improved wound contraction rate (excision wound, 99.62 0.59%; burn wound, 99.46%), breaking strength (393.2 g cm^2^), and granulation tissue weight (166.8 mg) [122]. According to the results of the burn wound healing model, the F2 poly-herbal ointment formulation has the greatest potential for healing burn wounds compared to the F-1 ointment formulation and plant extracts [83]. The mean VAS score for burning sensation decreased from 2.0238 to 1.3095 for Group I and from 1.4783 to 0.8913 for Group II. Group I had a reduction in lesion size from baseline of 0.781 mm to posttreatment, whereas Group II saw a reduction in lesion size from 0.939 mm to 0.439 mm [74].

Extract gel has been shown to decrease the size of an ulcer and speed up the healing process after trauma. The healing period was significantly shorter in the group using 75% red pomegranate extract gel compared to the group using 0.1% triamcinolone acetonide (*P*≤0.001) [82]. After three days of treatment, the average numbers of macrophages in the red pomegranate extract gel group, the triamcinolone acetonide 0.1% group, and the CMC-Na placebo group were 9, 16, and 22, respectively. The mean numbers of macrophages in the blood of people in the 5-day study groups were 27.2, 23.6, and 28.9 [84]. High quantities inhibited gingival fibroblast viability and migration, but low doses, without Zn (II), did not affect fibroblast vitality. Together, punicalagin and Zn (II) at low concentrations boosted fibroblast migratory velocity and distance [133]. Ulcer Inhibition % of *P. granatum* was 84.605 at a high dose of 500 mg/kg and 42.877 at a low dose of 250 mg/kg [85]. There was a statistically significant distinction between the two groups (*P*≤0.001). High levels of FGF-2 and TGF expression were observed in the treatment group after pomegranate extract was injected into their empty sockets [134]. In the incision wound model, 10% w/w punicalagin ointment showed the best wound healing strength at 201.83. On Day 15, in the excision wound model, the percent reduction in the wound was the highest, at 88; this was superior to the gold standard, which was 92% [95]. The extract significantly enhanced the material's breaking strength. Wound contraction and epithelialization both sped up significantly after being treated with the extract. Studies have shown that the extract's main chemical components, punicalagin, gallic acid, and ellagic acid, have a strong wound-healing effect [26]. The extract's strong antioxidant qualities have led to the hypothesis that there may be a connection between the improvement in histological wound healing and the decrease in oxidative stress in the wounded tissues [26].

In numerous models, *P. granatum* extracts have been shown to be quite beneficial for wound healing. The extracts demonstrated potential in material applications by improving the mechanical properties of treated membranes, including maximum tension, swelling index, and water vapor permeability. *P. granatum* extracts improved breaking strength, granulation tissue weight, and wound contraction rates in wound healing experiments as compared to control groups. Compared to traditional therapies such as triamcinolone acetonide, the 75% red pomegranate extract gel significantly reduced healing times and raised macrophage levels during the healing process. Furthermore, high-dose extracts demonstrated significant ulcer inhibition and increased wound healing strength, while punicalagin coupled with zinc boosted fibroblast migration and wound healing. Subsequent investigations ought to concentrate on elucidating the methods through which *P. granatum* extracts influence molecular and cellular functions. For the treatment of chronic wounds, we therefore propose that dressing membranes containing this extract are a promising product. Preclinical results must be confirmed in additional carefully planned clinical trials to ascertain the mechanism of action of the extract in the treatment of wounds. Additional clinical trials are required to confirm the safety and effectiveness of these extracts in people, as well as to evaluate their long-term effects and the best possible formulations. Standardization of extraction procedures is essential for reliable therapeutic results, and comparative studies should assess *P. granatum* extracts' efficacy in comparison to other therapies to determine the most advantageous uses for them.

#### 2.4.9. Antidiabetic Activity

Clinically and publicly, diabetes mellitus (DM) is a devastating disease. DM is a significant chronic metabolic disorder [91]. According to the WHO's report on worldwide statistics collected in 2020, around 422 million people around the world have diabetes; the majority of them live in low- and middle-income countries, and 1.6 million deaths are directly related to diabetes each year [16]. Unfortunately, modern medicine has yet to develop an effective treatment for diabetes. Treatment for diabetes includes injectable insulin and oral antidiabetic drugs [16]. There is a necessity to look for the most up-to-date antidiabetic drugs because oral hypoglycemic agents may cause various negative effects. Serum triglycerides were increased, and serum low-density lipoprotein cholesterol (LDL-C) was decreased in mellitus rats treated with 100 and 200 mg/kg methanolic extracts of *P. granatum* [16].

The ethanolic extract inhibited alpha-glucosidase most effectively, with IC_50_ values ranging from 53.34 to 15.18 U/L (100–1000 μg/mL) [30]. With an IC_50_ value of 603.50 μg/mL, ethanolic extract was shown to be the most potent inhibitor of lipase. The IC_50_ value for ACE inhibition by ethanolic extract was 519.45 μg/mL, and this value rose as the concentration ranged from 100 to 1000 μg/mL [30]. The 30%, 46%, 45%, and 39% inhibition rates observed with pomegranate rind and aril extracts from petroleum ether and dichloromethane were comparable to those observed with those from other sources [34]. In the hydroalcoholic and aqueous extracts, the inhibition of amylase had an IC_50_ of 9.804 and 19.011 mg/mL, respectively. At 50 mg/mL of glucose, yeast showed a strong inhibitory capacity for glucose absorption [35]. Blood glucose, triglycerides, serum cholesterol, LDL-C, AST, and ALT enzyme were all considerably lowered after 21 days of oral treatment of the fruit extract at doses of 150 and 300 mg/kg BW [87].

The results of the oral glucose tolerance test (OGTT), the insulin tolerance test (ITT), and the homeostasis model assessment of insulin resistance (HOMA-IR) all showed an increase in insulin sensitivity. Increased insulin-stimulated phosphorylation of insulin receptor substrate (IRS-1), Akt, and GSK-3 increased insulin signaling activity at the molecular level [89]. Diabetic rats treated with the extract (excluding Group 4 on Day 22) demonstrated a significant (*P*<0.05) reduction in blood glucose levels. However, plasma insulin levels significantly increased in diabetic rats treated with the extract (except for Group 4 on Day 22; *P*<0.05) [90]. Blood glucose, cholesterol, triglyceride, creatinine, and urea levels all fell precipitously when *P. granatum* (pomegranate) methanolic extracts were administered [91]. The IC_50_ values for pancreatic lipase (33.74 μg/mL), alpha-glucosidase (45.31 μg/mL), and alpha-amylase (43.24 μg/mL) were all significantly decreased when exposed to leaf extract [92]. Fasting blood sugar in the experimental group decreased more and hemoglobin levels rose higher than in the control group. In addition, fasting blood glucose levels decreased significantly (*P*<0.05) from Day 0 to Day 30. On Days 53 and 60, presumably due to pomegranate juice's lingering effects, similar results were recorded [93].

Diabetic rats exhibited decreased GSH and superoxide dismutase (SOD) along with elevated FBG, urea, BUN, creatinine, urine protein, malondialdehyde (MDA), and TNF. In addition to raising GSH and SOD, PGPE and sitagliptin treatment reduced SFBG, urea, BUN, creatinine, TNF, MDA, and total protein [94]. Mean serum glucose, triglyceride, cholesterol, ALT, and AST values were all found to be significantly higher in Group II diabetic control rats (*P*≤0.05). When compared to the diabetic control rats, all the treatment groups given pomegranate juice or pomegranate peel extract (Groups IV to VII) demonstrated statistically significant improvements in all of the parameters tested [95]. The findings also showed that diabetic rats had lower mean BW, RBC, and Hb levels than normal control rats (*P*≤0.05). In comparison to the diabetic control group by the end of the 45th day, all metrics significantly improved in the groups given metformin, pomegranate juice, and pomegranate peel extract (Groups III–V) [95].

This inhibitory ability can be explained by the fact that the hydroalcoholic and aqueous extracts include chemicals with functional groups like those of the substrate (starch), which occupies the enzyme's active site [35]. Polyphenols, alkaloids, terpenoids, and flavonoids have all been hypothesized to contribute to medicinal plants' antidiabetic action [91]. Sennosides A and B, as determined by pharmacological analysis, stimulate Auerbach's plexus in the gastrointestinal system without forming a strong interaction with the active site of the pancreatic lipase enzyme [92]. Another probable mechanism is that blood macrophages suffer apoptosis after being exposed to crude extracts, which triggers the release of secretory insulin. Conversely, hypoglycemia in the brain will signal the release of insulin when dendritic cells in the brain are stimulated. However, insulin resistance and DM are likely exacerbated by fat covering the insulin receptor and blocking the receptor's ability to connect with cells and trigger insulin release. It depicts a state with a high prevalence of diabetic complications. Damage to the nerve system (nephron damage) is the end outcome of nephropathy, retinopathy, and neuropathy.

#### 2.4.10. Anticancer Activity

The search for and development of medicines made from plant-based raw materials has long been one of the most important aspects of human knowledge. After 48 h of incubation, the acetonic fraction of pomegranate peels showed the greatest antiproliferative efficiency against MCF7 cancer cells with an IC_50_ of 8.15 μg/mL. In contrast, the methanolic extract was highly selective against transformed cancer cells in comparison to the normal cell line with a selectivity index of 5.93 [29]. The results showed that the MCF-7 breast cancer cell line was the most susceptible to the peel extract of both domestic and imported *P. granatum*, whereas the estrogen-negative MDA-MB 231 cell line was the least susceptible [116]. As indicated, the results demonstrated that AgNPs dramatically reduced the viability of human cervical cancer cells. The growth capacity of HeLa cells was effectively inhibited by the AgNPs; therefore, samples at 50, 100, 150, 200, and 250 μg/mL were collected for future study [97]. Silver nanoparticles produced from *P. granatum* were effective against MCF-2, PC3, A-549, HeLa, and HepG2 cell lines, with IC_50_ values of 108.7 and 88.42 μg/mL for PC-3 and A 549 cancer cell lines, respectively [98]. The extract had an IC_50_ of 130,914 μg/mL for MDA-MB231 and 133.77 μg/mL for HT29, respectively [99]. Chondrocytes treated with the extract exhibit dose-dependent increases in proliferation and decreases in apoptosis because of lower oxidative stress. At higher extract doses, the number of chondrocytes increased significantly (*P*<0.05) compared to indomethacin (positive control) treated cells [135].

The study demonstrates that *P. granatum* and its derivatives have strong anticancer effects. The antiproliferative impact against MCF7 breast cancer cells was best in the acetonic fraction of pomegranate peels, with an IC_50_ of 8.15 μg/mL. In contrast, the methanolic extract had a significant selectivity for cancer cells compared to normal cells. In contrast to the estrogen-negative MDA-MB 231 cell line, the MCF-7 cell line showed a marked increase in sensitivity to both local and imported pomegranate peel extracts. In addition, various cancer cell lines, such as MCF-2, PC3, A-549, HeLa, and HepG2, were effectively reduced in viability by silver nanoparticles produced by *P. granatum.* In addition to lowering oxidative stress better than indomethacin, the extracts showed an increase in chondrocyte proliferation and a decrease in apoptosis that was dosage dependent.

The precise pathways by which nanoparticles and *P. granatum* extracts exert their anticancer properties should be further investigated in future studies. To determine the practicality and security of these nanoparticles and extracts for the treatment of cancer in humans, additional research is required. Improving their therapeutic potential could also be achieved by refining extraction procedures and formulating nanoparticles.

#### 2.4.11. Neuroprotective Activity

Neuroprotective is a major global public health concern, which is generally acknowledged. Despite the high frequency, morbidity, and mortality rates of these illnesses, the medical care that is now provided for them is insufficient. It has long been thought that medicinal plants, especially those with a long history of traditional use, hold a lot of untapped potential in the form of fresh, strong medications that could help treat liver disorders. Longevity extension, enhanced locomotor skills, and rescue of neurodegeneration in the ommatidia of A42-expressing *Drosophila* showed that the leaves may greatly mitigate the detrimental morphological alterations from A42 protein in *Drosophila*. Potential in vivo neuroprotective effects of the extracts against beta-amyloid-induced neurotoxicity in *Drosophila melanogaster* [100]. There was a significant reduction in the hepatic indicators of doxorubicin toxicity (ASAT, ALAT, and ALP), as well as total protein and albumin, after pretreatment with the extract [101]. MDA and creatine kinase (CK)-MB enzyme activities were both significantly decreased before and after treatment with the extract. The antioxidant enzymes glutathione peroxidase (GPx), catalase (CAT), and SOD were also elevated [102]. The findings indicated that the use of vancomycin led to significant liver and kidney damage, evidenced by elevated levels of tissue injury markers, lipid oxidation (MDA), and inflammation markers (C-reactive protein), as well as reduced levels of antioxidants and proteins [136]. After taking seed extracts orally for 12 weeks, DEN-induced changes in serum AFP, bilirubin, triglycerides, cholesterol, and urea were significantly reversed. In terms of chromosomal aberration rates and micronucleated polychromatic erythrocytes, the observations showed that the MMC group had the highest levels; this rate decreased with 150 mg/kg PG and 300 mg/kg PG peel extract application with MMC, and the dose-dependent increase in this decrease was statistically significant (*P*<0.001) [8]. Acute CCl_4_-treated animals exhibited extensive macro and microvesicular steatosis, mononuclear inflammatory cell infiltrations in the portal area and parenchyma, and necrotic alterations, while chronic CCl_4_-treated animals exhibited mild to severe fibrosis with lobulation formation on top of acute findings [137]. The proposed mode of action involves antioxidative activities against CCl_4_-induced oxidative stress by lowering the levels of MDA and NO, which reflect the seriousness of liver damage in acute and chronic hepatotoxicity [137].

#### 2.4.12. Other Activities

Anti-anxiety: In around three to 5 minutes, the typical animal will slip and fall off the rotarod device. Group 4 (100 mg/kg) experienced a marked decrease in falling time because of motor incoordination. Open-arm entry times lengthen with extract dose 2 (Group 4) [110]. Antidandruff: Alcoholic extracts at a concentration of 3% showed significant antidandruff efficacy against the fungi used in the study. The alcohol extract contained maltol, a major phytoconstituent [77].

Anti-gingivitis: There were no reported side effects; therefore, pomegranate peel extract shows promise as an alternate treatment for chronic gingivitis, at least within the scope of this trial [108].

Mental health: After 120 min, a 100 mg/mL concentration of *P. granatum* peel extract showed the highest scolicidal efficacy [109]. Bone healing: Compared to the C and GS groups, the GS/PSO group's bone defects exhibited much higher evidence of bone growth, mineralization, and maturation. With the exception of the C/GS and GS/GS/PSO groups at 4 weeks, significant variations among all groups were observed for bone cells at both 2 and 4 weeks [125].

Antihypertensive activity: Hypertensive rats given salt fludrocortisones had their blood pressure significantly dropped by the extract, but normotensive rats showed no changes to their average blood pressure or heart rate [111].

Anti-asthma activity: It demonstrated additive anti-inflammatory effects in reducing gene expression of CXCR1, CXCR2, IL-6, and IL-8 in white blood cells exposed to LPS. LPS-exposed cells also showed enhanced IL-10 gene expression and decreased NO and TNF. Anti-dyslipidemia activity: Two weeks of treatment with 100 mg/kg resulted in a 27.6% and 34.7% reduction in total cholesterol and LDL-C levels, respectively. Total cholesterol and LDL-C levels were reduced by up to 59.9% and 75.05%, respectively, after 4 weeks of therapy with the extract [112].

Anti-post-surgical activity: The adhesion area and severity of adhesions in the control group rats were statistically higher than in any of the experimental groups. Foreign body reactivity in the serosal layer was also significantly lower in the EG-400 group compared to the other three groups [113].

Postmenopausal syndrome activity: Animals given a combination of extracts from four medicines responded differently depending on the dose. There was a notable rise in uterine weight, bone mineral density (BMD), and femur hardness. Serum calcium and phosphorus levels were also found to be higher than in the OVX control group, whereas urinary calcium and phosphorus levels were significantly lower. The extracts' protective effect is supported by histological findings [114].

Aphrodisiac activity: Oral administration of 1500 mg/kg of pomegranate extract significantly increased sexual behavior in male rats. There was no statistically significant difference in the mounting rate, intromission rate, mounting latency, intromission latency, ejaculation latency, or post-ejaculation interval. The frequency of increases was substantially different among the three groups (control, pomegranate crude extracts, and sildenafil). Furthermore, there was no discernible difference in extract efficacy between the placebo, pomegranate crude extracts, and sildenafil. Significant differences in testosterone levels were seen between the pomegranate, sildenafil, and control groups. Alopecia: Extract gel at a concentration of 20%w/v ethanolic was found to be effective in a study of people with alopecia [138].

Antiarthritic activity: Increases (*P*< 0.001) in RBCs and Hb and decreases (*P*< 0.001) in WBCs and ESRs were seen at a dose of 75 mg/kg [104]. Anticoccidial activity: Oocyst output was reduced (92.8), bird weight was increased (1403 g), and feed conversion ratio was increased (1.66), all of which pointed to the methanolic extract of the fruit peel of *P. granatum* having the greatest effect [105]. The results confirmed reduced cecal inflammation, increased immunoglobulin expression in cecal tissue, improved cecal integrity, and restoration of its REDOX state through antioxidant testing, ELISA for anticoccidial mechanisms, and pathological observations [106].

Osteoporosis is a progressive bone disease that causes bones to become brittle and break easily from mild trauma. This is because bone mass decreases because of the condition [107]. Although many synthetic agents have been developed to treat osteoporosis, including estrogens in hormone replacement therapy and selective estrogen receptor modulators like raloxifene, droloxifene, bisphosphonates, and calcitonin, they are all linked to unpleasant side effects like hypercalcemia, hypercalciuria, an increased risk of endometrial and breast cancer, breast tenderness, menstruation, thromboembolic events, vaginal bleeding, and hot flashes [107]. Therefore, investigating naturally occurring compounds, particularly those derived from plants, that may prevent bone loss and have no negative side effects would be the most beneficial option. The femoral length, weight, volume, and density were all raised in the treatment groups, as was the fourth lumbar hardness (*P* < 0.001). Biochemical markers also showed statistical significance [107].

#### 2.4.13. Toxicity

Worldwide, although mostly in less developed regions, people use medicinal herbs. They are especially popular because they are readily available and inexpensive in the area. People everywhere assume that plant-based medicines are risk-free since they are natural. The information implies differently, though. They can be quite dangerous if the incorrect ones are chosen, or they are improperly cooked. So, it is important to assess the safety of plant extracts. Numerous compounds derived from medicinal plants have been shown to have positive biological effects on researchers. All animals monitored 24 h after receiving oral doses of 250 or 500 mg/kg of pomegranate fruit peel methanolic or aqueous extracts showed no signs of death [46].

The MTT assay demonstrated that cell viability was greater than 80% across the full concentration spectrum of HEPg (125–500 μg/mL). The percentage of alive cells dropped to almost 70% at 1000 ng/mL HEPg. *P. granatum* extracts were efficient against the microorganisms at low cytotoxic doses [60]. There was no evidence of toxicity up to 500 mg/kg for the butanolic fraction [104]. Throughout the experiment, we saw no signs of toxicity or fatality [131]. According to an acute oral toxicity investigation, *P. granatum* crude extracts are safe at doses up to 2000 mg/kg. Because no chickens died in any of the five test groups, LD_50_ could not be calculated [105]. After 24 h of extract administration, no acute oral toxicity symptoms were seen. High-dose oral dosing (up to 2000 mg/kg) for 21 days (once daily) resulted in no signs of toxicity or mortality. This shows that using the extract for an extended period is safe [90].

There were no deaths or drug-induced physical symptoms seen during the acute toxicity testing of the extracts. From the beginning of the treatment through the finish, no fatalities or adverse reactions were seen at any of the chosen doses [91]. Both 2500 and 2000 ppm were shown to be harmless [100]. Normalization of renal function measures and amelioration of histological abnormalities show that co-administration of the extract with gentamicin protects kidneys from the nephrotoxic effects of gentamicin [115]. The WT groups showed no mortality, no behavioral abnormalities, and no significant changes in microhematocrit, serum biochemical markers, internal organ histology, or oxidative stress [139].

The findings of this study indicate that there are no adverse health effects associated with consuming excessive amounts of fruit peel extract. Toxicology testing is the starting point for any pharmaceutical or herbal formulation. Because of this, we cannot say with certainty that *P. granatum* is safe or harmful based on the available toxicological research. It is recommended that additional thorough research of the plant and its components is necessary to determine their toxicity.

### 2.5. Clinical Trial Studies

We may conclude that *P. granatum* has beneficial effects on a variety of illnesses based on the findings of clinical research (Table 3). The results demonstrate improved blood circulation and erectile responsiveness in erectile dysfunction patients. According to research, pomegranates can enhance sperm motility and density, two characteristics of semen. By lowering the levels of damage markers, it may also improve liver function and lessen symptoms associated with uterine leiomyoma. Additionally, because it lowers cholesterol, it has been shown to be good for the heart and helps prevent kidney stones. It is recorded to be in the management of the menstrual cycle and reducing postmenopausal symptoms. According to the clinical trials, it also aids in the treatment of knee osteoarthritis and the symptoms of polycystic ovary syndrome (PCOS). It also counters inflammations that exist in rheumatoid arthritis and inflammatory bowel disease patients. However, this gives promise to further study these effects and to examine the best dosage of *P. granatum* extract. Nonetheless, more controlled large-scale clinical trials are needed for all the conditions to determine the effectiveness and risk benefit profile of *P. granatum* extract for these diseases.

## 3. Conclusion

Natural resources provide a wealth of materials that could be mined for the creation of cures for a variety of chronic diseases. There is scientific evidence for the pharmacological action of numerous medicinal plants and their compounds. In this study, we looked at the evidence that *P. granatum* has medicinal and pharmacological effects upon evaluation of the included articles. Many cultures employ *P. granatum* as a traditional medicine, and the plant's pharmacological qualities have been studied. This review gives a thorough synopsis of the relationship between pomegranate traditional medicine and contemporary research. It elucidated the positive function of pomegranate in health care prevention, which is both scientifically grounded in current preventive medicine and consistent with the idea of “preventive treatment of disease” in traditional medicine. Studies have mostly focused on the plant extract rather than distinct metabolites. Study undertaken in vitro and in vivo provides proof that it has a supportive role in preventing and treating many diseases. *P. granatum* exhibits substantial potential as an antioxidant, anti-inflammatory, analgesic, and antimicrobial agent and has antidiabetic and neuroprotective effect. High levels of flavonoid and polyphenol chemicals in the fruit peel were responsible for its impressive biological activity. It might be used as a new method for treating ailments. Understanding each bioactive chemical's role and process as well as the potential therapeutic effects that lead to the development of new medications is essential. This variability in preclinical and clinical trials could be attributed to the extraction methods, plant parts used, plant age, and the collection location, all of which significantly influence the types of phytochemicals present in the plant. Different solvents used in extraction may produce different levels of bioactive compounds owing to solubility and ability of the plant extract. Nonetheless, more controlled large-scale clinical trials are needed for all the conditions to determine the effectiveness and risk benefit profile of *P. granatum* extract for these diseases. There has not been examination of the molecular mechanisms of action of the extract or pure compounds. More controlled large-scale clinical trials are needed for all the diseases to determine the effectiveness and risk benefit profile of *P. granatum* extract.

## Figures and Tables

**Table 1:** Database search strategy.

Database	Keywords
ScienceDirect	“Anticancer” “anti-cancer” “anticancer of *Punica granatum” “anti*-microbial” “Pomegranate” “*Punica granatum 2018” “P. granatum”* “antimicrobial*”* “antibacterial” “antiinflammation” “antiviral” “hypertension”
PubMed	“Antidiabetes mellitu” “Pomegranate” “GCMS” “HPLC” “anti-cancer” “*Punica granatum” “a*nti-anxiety” “bone healing” “wound healing” “antifungal” “anti-obesity” “anti-inflammation” “Aphrodisiac”
Wiley	*“P. granatum”* “anticancer” antiviral” “anti-cancer” “anticancer of *Punica granatum” “anti*-microbial” “Pomegranate” “*Punica granatum 2018”* “antimicrobial*”* “antibacterial” “antiinflammation” ““hypertension”
Google Scholar	“Anticancer” “anti-cancer” “anticancer of *Punica granatum” “anti*-microbial” “Pomegranate” “*Punica granatum 2018” “P. granatum”* “antimicrobial*”* “antibacterial” “antiinflammation” “antiviral” “hypertension” “antidiabetes mellitu” “Pomegranate” “GCMS” “HPLC” “anti-cancer” “*Punica granatum” “*Anti- Anxiety” “Bone healing” “Wound healing” “antifungal” “anti-obesity” “anti-inflammation” “Aphrodisiac”
Hindawi	*“P. granatum”* “anticancer” antiviral” “anti-cancer” “anticancer of *Punica granatum” “anti*-microbial” “Pomegranate” “*Punica granatum 2018”* “antimicrobial*”* “antibacterial” “antiinflammation” ““hypertension”
Springer	“*Punica granatum” “P. granatum”* “antimicrobial” “antibacterial” “antivirus” “antifungal” “anti-microbial” “anti-fungal” “anti-cancer” “antioxidant” “antioxidant” “anti-anxiety” “HPLC” “GCMS” “Pomegranate extract”

**Table 2:** Demographic profile of the documented biological studies.

S/N	Activity	Method	Parts	Solvents	Concentrations	References
1	Antioxidant	DPPH	Fruits	Aqueous	0.1 and 15 mg/mL	[26]
		DPPH	Fruits	Aqueous	1000 μg/mL	[19]
		DPPH, ABTS	Leaves	Methanol	NA	[27]
		DPPH, ABTS	Leaves	Hexanic, ethyl acetate, and aqueous fractions	NA	[28]
		DPPH	Fruits	Aqueous, methanol, acetone, hexane	1 mg/mL	[29]
		DPPH	NA	Aqueous, ethanol	100–1000 μg/mL	[30]
		DPPH	Fruits, seeds	Aqueous, ethanol	NA	[31]
		ABTS, DPPH	Peel, pulp, and seeds	Aqueous	NA	[32]
		In vivo	NA	NA	NA	[33]
		DPPH	NA	Aqueous	NA	[34]
		FRAP, H_2_ O_2,_ DPPH	NA	NA	NA	[35]
		DPPH	Fruits	NA	25, 50, 100, 250 and 500 μg/mL	[36]
		DPPH	NA	NA	NA	[37]
		In vivo	Fruits	Ethanol	1000, 1500 and 2000 ppm	[38]
		DPPH, ABTS	Flower	Acetone, aqueous		[39]
		DPPH	Fruits	NA	NA	[40]
		DPPH, ABTS	Fruits	NA	NA	[41]

2	Anti-inflammatory activity	In vivo	Flower	Methanol, aqueous		[42]
		In vivo	Fruit	NA	NA	[43]
		In vitro LOX inhibition assay	Fruit	80% methanol	NA	[44]
		In vivo	Rind	NA	20, 40, 80 mg/kg BW	[45]
		In vivo	Fruits	Methanol, aqueous	250 and 500 mg/kg	[46]

3	Antibacterial activity	Ager well	Stem bark	Hexane	50, 25, 12.50, and 6.25 μg/mL	[47]
		Ager well	Fruits	Aqueous, ethanol, chloroform	NA	[48]
			Fruits	Methanol	NA	[49]
		Ager well method	Leaves	Ethanol	5120 μg/mL	[49]
		MIC	Fruits	NA	1, 10, and 100 μg/mL	[50]
		Disc	Seeds, fruit	Ethanol	NA	[51]
			Leaves	Hexanic, ethyl acetate, and aqueous fractions	NA	[28]
		Disc	Fruit	NA	NA	[52]
		Disc	Fruits	Aqueous, methanol, acetone, hexane	10 mg/disk	[29]
		In vivo	NA	NA		[53]
			NA	NA	200 mg/mL	[54]
		Disc	Fruits, seeds	Aqueous, ethanol		[31]
		Ager well	Fruits	Ethanol	100, 50, 25 mg/mL	[55]
			Seeds	NA	NA	[56]
		In vivo	NA	Ethyl acetate fraction	100, 200, 400 mg/kg	[57]
			NA	Essential oil	200, 400, 600, and 800 μL/L	[58]
			Fruits	Aqueous	NA	[59]
		Broth assays	Leaf	NA	NA	[60]
		Checkerboard method.	Fruits	50% ethanol	NA	[61]
		Disc	Fruits	NA	NA	[62]
			Fruits	NA	25, 50, 100, 250 and 500 μg/mL	[36]
		Microdilution Assay	NA	NA		[63]
		MIC	Flower	Methanol, ethanol, aqueous	13,000, 6500, 3250, 1625, and 812.5 μg/mL	[15]
		Disc	Flower	NA	NA	[64]
			Fruits	NA	NA	[65]
		NA	NA	NA	NA	[66]
			Fruits	Ethanol	NA	[67]
		In vivo		NA	NA	[68]
		Disc	Seeds	NA	100, 250, 500 and 1000 ppm	[69]
		NA	NA	Methanol, aqueous	NA	[70]
		NA	Bark	Aqueous	NA	[71]
		Disc	Fruits	NA	NA	[72]
		Ager well	Fruits	NA	5 and 10 mg/mL	[73]
		In vivo	NA	NA	0.2%	[74]

4	Anthelmintic activity	Adult motility assay, egg hatch inhibition assay	Stem, flower leaves	Methanol	10, 5, 2.5, and 1.25 mg/mg/0.1, 0.25, 0.5, and 1 mg/mL	[20]
		NA	NA	Ethanol	25, 50, 75 mg/mL	[75]
		In vivo	NA	Aqueous	NA	[76]
		In vivo	Leaves	Ethanol, aqueous	NA	[77]
		In vivo	NA		50, 100, 150 mg/mL	[78]
			Fruits	Ethanol	100, 150, 200 mg/mL	[79]
		In vivo	NA	Aqueous	50,100 and 150 mg/mL	[80]

5	Antiviral activity		Leaves	Aqueous		[81]

6	Wound healing activity	In vivo	Fruits	Aqueous	1%	[26]
		In vivo	Fruits	NA	12.5%, 25%, 50% and 75%	[82]
		In vivo	Bark	Petroleum ether	10% & 15% (w/w)	[83]
		In vivo	NA	NA	75%	[84]
		In vivo	Fruit	Aqueous	250 mg/kg, 500 mg/kg	[85]
		In vitro excision and incision models	Fruits	Ethanol	10% w/w	[86]

7	Antidiabetic activity	In vivo	Fruits	Aqueous	150 and 300 mg/kg bw	[87]
		In vivo	Flower	Methanol	100 and 200 mg/kg bw	[88]
		In vivo	Flower	Ethanol	50 and 100 mg/kg	[89]
		*α*-Glucosidase		Ethanol, aqueous		[30]
		In vivo	Leaves	NA	100, 200, 400, and 600 mg/kg	[90]
		In vivo	NA	NA	100 and 200 mg/kg	[16]
		In vivo	Fruits	Aqueous	500 mg	[91]
		In vivo	Leaves	Ethanol	NA	[92]
		In vivo	Fruits	NA	NA	[93]
		In vivo	Fruits	Aqueous	200 mg/kg/day	[94]
		In vivo	NA	NA	100 mg/day	[95]
		In vivo	Fruits	Aqueous methanol (40:60)	NA	[96]
		NA	NA	Ethanol, aqueous	NA	[30]

8	Anticancer activity		Fruits	Aqueous, methanol, Acetone, hexane	10 mg/mL	[29]
		MTT	Leaves	Aqueous	50, 100, 150, 200 and 250 μg/mL	[97]
		MTT	Fruits	Methanol	NA	[98]
		MTT	Seeds	NA	10–250 μg/mL	[99]

9	Neuroprotective activity	In vivo	Leaves	Chloroform, ethanol	NA	[100]
		In vivo	Fruit	72% ethanol	100 mg/kg	[101]
		In vivo	Fruits	Ethanol	100 mg/kg	[102]
		In vivo	Seeds	NA	400 mg/kg	[103]
		In vivo	NA	NA	150, 300, 150 mg/kg	[8]

*Other biological activities*						
10	Antiarthritic activity	In vivo	Fruits	Butanol Fraction	50 and 75 mg/kg body weight.	[104]

11	Anticoccidial activity	In vivo	Fruits	Hexane, methanol, aqueous	2000 mg/kg body weight	[105]
		In vivo	Fruits	NA	NA	[106]

12	Antidandruff	In vivo	Leaves	Ethanol, aqueous	NA	[77]

13	Antiosteoporosis activity	In vivo	NA	NA	100, 300, and 500 mg/kg body	[107]

14	Anti-gingivitis	In vivo	Fruits	NA	5%	[108]

15	Anti-scolicidal	In vivo	Fruit	Aqueous	100, 10, and 1 mg/mL	[109]

16	Anti-anxiety	In vivo	Leaves	Methanol, ethanol	50, 100 mg/kg	[110]

17	Bone healing	In vivo	Seed	NA		[72]

18	Antihypertensive	In vivo	Fruits	Ethyl Acetate	200 mg/kg and 400 mg/kg	[111]

19	Anti-asthma	In vivo	Fruits	Methanol		[33]

20	Anti-dyslipidemia	In vivo	Leaves	Ethanol	100 mg/kg	[112]

21	Anti-post-surgical peritoneal adhesions	In vivo	Flower	Ethanol aqueous (70:30)	100, 200, and 400 mg/kg/day	[113]

22	Postmenopausal syndrome	In vivo	Fruit	Ethanol	2000, 3000 and 5000 mg/kg bw	[114]

23	Toxicity	In vivo	Fruits	Butanol Fraction	500 mg/kg	[104]
		In vivo	Leaves	Methanol	100, 200 and 400 mg/kg p.o.	[115]
		In vivo	Fruits	NA	2000 mg/kg body weight	[105]
		In vivo	Bark	Petroleum ether	10% & 15% (w/w)	[83]
		Brine shrimp lethality bioassay	Leaves	Chloroform, ethanol	2500 and 2000 ppm	[100]
		In vivo	Leaves	NA	100, 200, 400, and 600 mg/kg	[90]
		MTT	Leaves	NA	NA	[60]
		In vivo	Fruits	Aqueous	NA	[91]
		In vivo	Fruits	Methanol, aqueous	250, 500 mg/kg doses	[46]

*Note:* ABTS = 3-ethyl-benzothiazoline-6-sulfonic acid, IC_50_ = concentration causing 50% inhibition, MTT = 3,-4, 5 dimethylthiazol-2,5 diphenyl tetrazolium bromide assay, H_2_ O_2_ = hydrogen peroxide scavenging activity, and PAgNPs = green synthesis of silver nanoparticles.

Abbreviations: ALT = alanine aminotransferase, AST = aspartate aminotransferase, bw = body weight, DPPH = 2,2-diphenyl-1-picrylhydrazyl, FRAP = ferric reducing antioxidant power, Hb = hemoglobin, NA = not available, RBC = red blood cell, T. S = transverse section, WBC = white blood cell, and ZOI = zone of inhibition.

**Table 3:** Clinical trial studies of *Punica granatum*.

S/N	Diseases	Plant part	Sample size	Duration (days)	Summary of major findings	References
1	Erectile dysfunction	Fruit (juice)	60	14	Twenty-five participants said they felt better after consuming pomegranate juice. This pilot trial did not reach statistical significance overall, but it raises the prospect that bigger cohorts and longer treatment durations could.	[140]

2	Improvement of semen quality	Fruit (extract)	70	90	There was a 62% rise, from 23.4 million to 37.8 million, in the average total number of motile sperm after 3 months of active treatment. The outcome of the treatment on sperm morphology was unaffected.	[141]

3	Uterine leiomyoma	Flower (syrup)	19	78	Over the course of the trial's 3 months, the average size of uterine fibroid dropped by 16.1%. The size of the uterus and the bleeding caused by leiomyomas can be reduced with the use of syrup.	[142]

4	Liver diseases	Fruit (juice)	65	84	Pomegranate juice may be an effective way to boost the antioxidant status of people with nonalcoholic fatty liver disease (NAFLD), as the study found that liver enzyme levels and body mass index (BMI) had dropped considerably at the end.	[143]

5	Nephrolithiasis	Fruit (juice)	30	90	Supplementation with 1000 mg of pomegranate polyphenol extract daily may provide minor benefits in reducing calcium oxalate supersaturation.	[144]

6	Atherosclerotic cardiovascular disease	Fruit (juice)	101	365	The group that consumed fruit showed a considerable improvement in systolic blood pressure, pulse pressure, triglycerides, and HDL level as measured in time, while the group that took placebo showed no such improvement.	[145]
		Fruit (juice)	101	365	Consumption of pomegranate juice over an extended period of time improves nontraditional risk factors for cardiovascular disease, slows the development of atherosclerosis, boosts innate immunity, and ultimately decreases morbidity in hemodialysis patients.	[146]
		Fruit (juice)	41	56	Fruit consumption showed significant effect on blood pressure, serum triglycerides, high-density lipoprotein cholesterol, oxidative stress, and inflammation in individuals undergoing hemodialysis.	[147]
		Fruit (juice)	24	84	Throughout the trial duration, neither treatment significantly influenced lipid profiles, plasma C-reactive protein, interleukin 6, F2-isoprostane or isofuran concentrations, predialysis systolic or diastolic blood pressure, nor altered monocyte cytokine production levels.	[148]
		Fruit (juice)	27	90	The consumption of fruit juice mitigated the rise in systemic oxidative stress and inflammation caused by intravenous iron administration during the dialysis session.	[149]
		Fruit (juice)	33	183	Fruit extract may lower blood pressure and enhance antioxidant activity in hemodialysis patients; however, it did not improve other cardiovascular risk factors, physical function, or muscle strength.	[150]

7	Central precocious puberty (CPP)	Fruit (extract)	225	90	In Chinese girls with polycystic ovary syndrome (PCOS), taking pomegranate extract daily enhanced the efficacy of gonadotropin-releasing hormone (GnRH) analog treatment.	[151]

8	Postmenopausal	Seed (oil)	81	84	In postmenopausal women, seed oil does not markedly diminish hot flashes after a 12-week observation period; nevertheless, additional research is required to explore the long-term effects.	[152]

9	Menstrual bleeding	Flower	94	5	The extract decreased hemorrhage. The mean (SD) score of the pictorial blood loss assessment chart (PBAC) decreased from 304.92 (176.17) and 304.44 (192.72) to 164.60 (100.24) and 143.13 (96.07) during the third treatment cycle, respectively (*P*< 0.001).	[153]

10	Polycystic ovarian syndrome	Fruit (juice)	92	212	Significant insulin resistance and sensitivity changes occurred. BMI, weight, and waist circumference dropped considerably. Testosterone levels dropped substantially.	[154]

11	Polycystic ovarian syndrome	Fruit (juice)	92	56	Participants saw improvements in their blood pressure, inflammation, oxidative stress, and lipid profiles during that period.	[155]

12	Knee osteoarthritis	Peel	60	56	It has been exhibited that peel extracts may have a positive effect on symptoms in women with knee joint pain due to inflammation relief and antioxidant content.	[156]

13	Rheumatoid arthritis	Fruit (extract)	8	84	Fruit extract decreased the Disease Activity Index (DAS28) in patients with rheumatoid arthritis.	[157]
		Fruit (extract)	55	56	Fruit extract mitigates disease activity and enhances certain blood indicators of inflammation and oxidative stress in patients with rheumatoid arthritis.	[158]
		Fruit (juice)	36	42	Consumption of the fruit juice enhanced physical function and reduced stiffness, diminished cartilage-degrading enzymes, and elevated antioxidant levels in people with knee conditions.	[159]

14	Nausea and vomiting	Fruit (syrup)	74		The efficacy of pomegranate in alleviating nausea and vomiting during pregnancy was validated in the syrup group compared to the control group, demonstrating significant differences.	[160]

15	Ulcerative colitis	Peel (extract)	78	28	The colitis activity index experienced a considerable reduction.	[161]
		Peel (extract)	62	28	The Lichtiger Colitis Activity Index score decreased (−1.68 ± 3.85, *P* = 0.019), and the clinical response was elevated.	[162]

16	Appetite stimulant	Fruit (syrup)	100	60	The intervention group had enhanced abdominal fullness, increased appetite, and greater satiety compared to the placebo syrup.	[163]

17	Inflammatory bowel disease		32	28	Herbal preparation showed no greater efficacy in alleviating diarrhea-predominant irritable bowel syndrome symptoms.	[164]
		Fruit (juice)	36	84	Fecal calprotectin levels decreased after the ingestion of pomegranate juice.	[165]

## Data Availability

Data are available within the article.

## Nomenclature

ABTS: 2,2′-Azino-bis(3-ethylbenzothiazoline-6-sulfonic acid)
AgNPs: Silver nanoparticles
DM: Diabetes mellitus
DNA: Deoxyribonucleic acid
DPPH: 2,2-Diphenyl-1-picrylhydrazyl
GCMS: Gas chromatography and mass spectrometry
FRAP: Ferric reducing antioxidant power
HHV-3: Human herpesvirus 3
IC_50_: Half maximal inhibitory concentration
MIC: Minimum inhibitory concentration
MBC: Minimum bactericidal concentration
MRSA: Methicillin-resistant *S. aureus*
MFC: Minimum fungicidal concentration
MTT: Tetrazolium bromide method
PCOS: Polycystic ovary syndrome
qPCR: Quantitative polymerase chain reaction
RNA: Ribonucleic acid
RBC: Red blood cell
WHO: World Health Organization
WBC: White blood cell
ZOI: Zone of inhibition
